# 2-(3-Eth­oxy-2-hy­droxy­benz­ylidene)-*N*-phenyl­hydrazinecarboxamide

**DOI:** 10.1107/S1600536811041857

**Published:** 2011-10-22

**Authors:** M. Sithambaresan, M. R. Prathapachandra Kurup

**Affiliations:** a Department of Chemistry, Faculty of Science, Eastern University, Sri Lanka, Chenkalady, Sri Lanka; b Department of Applied Chemistry, Cochin University of Science and Technology, Kochi 682022, India

## Abstract

The title compound, C_16_H_17_N_3_O_3_, exists in the *E* configuration with respect to the azomethine double bond. The mol­ecule is close to planar, with a dihedral angle of 6.7 (1)° between the aromatic rings. The phenolic O atom functions as donor and acceptor by forming intramolec­ular O—H⋯O and inter­molecular N—H⋯O hydrogen bonds, respectively. Two-dimensional packing is fashioned through an inter­molecular hydrogen bonding network in an offset manner.

## Related literature

For background to *N*-phenyl­hydrazinecarboxamides and their complexes, see: Reena *et al.* (2008 ▶). For the synthesis of related compounds, see: Siji *et al.* (2010 ▶). For related structures, see: Kayed *et al.* (2011 ▶); Kala *et al.* (2007 ▶); Kurup *et al.* (2011 ▶); Reena & Kurup (2010 ▶).
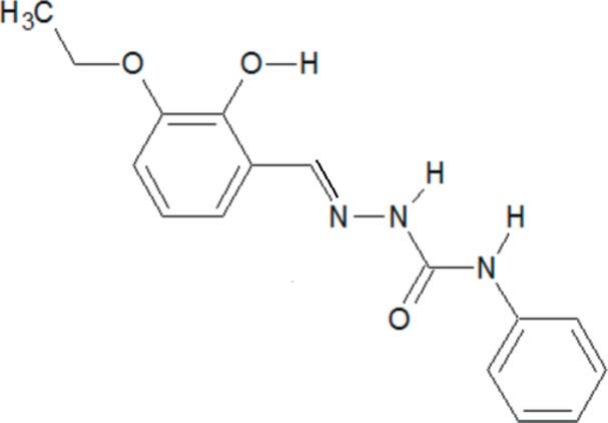


## Experimental

### 

#### Crystal data

C_16_H_17_N_3_O_3_
*M* *_r_* = 299.33
Monoclinic, 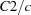

*a* = 30.1352 (13) Å
*b* = 5.5552 (3) Å
*c* = 18.2232 (8) Å
β = 92.753 (2)°
*V* = 3047.2 (2) Å^3^
*Z* = 8
Mo *K*α radiation
μ = 0.09 mm^−1^
*T* = 296 K
0.50 × 0.30 × 0.10 mm

#### Data collection

Bruker APEXII CCD diffractometer
Absorption correction: multi-scan (*SADABS*; Sheldrick, 1996) ▶ *T*_min_ = 0.967, *T*_max_ = 0.991
10811 measured reflections
2687 independent reflections
2066 reflections with *I* > 2σ(*I*)
*R*_int_ = 0.035

#### Refinement

*R*[*F* ^2^ > 2σ(*F* ^2^)] = 0.040
*wR*(*F* ^2^) = 0.137
*S* = 1.06
2687 reflections
213 parameters
2 restraints
H atoms treated by a mixture of independent and constrained refinement
Δρ_max_ = 0.16 e Å^−3^
Δρ_min_ = −0.13 e Å^−3^

### 

Data collection: *APEX2* (Bruker, 2004 ▶); cell refinement: *SAINT* (Bruker, 2004 ▶); data reduction: *SAINT*; program(s) used to solve structure: *SHELXS97* (Sheldrick, 2008 ▶); program(s) used to refine structure: *SHELXL97* (Sheldrick, 2008 ▶); molecular graphics: *SHELXTL* (Sheldrick, 2008 ▶) and *ORTEP-3* (Farrugia, 1997 ▶); software used to prepare material for publication: *SHELXL97* and *publCIF* (Westrip, 2010 ▶).

## Supplementary Material

Crystal structure: contains datablock(s) global, I. DOI: 10.1107/S1600536811041857/ng5242sup1.cif

Structure factors: contains datablock(s) I. DOI: 10.1107/S1600536811041857/ng5242Isup2.hkl

Supplementary material file. DOI: 10.1107/S1600536811041857/ng5242Isup3.cml

Additional supplementary materials:  crystallographic information; 3D view; checkCIF report

## Figures and Tables

**Table 1:** Hydrogen-bond geometry (Å, °)

*D*—H⋯*A*	*D*—H	H⋯*A*	*D*⋯*A*	*D*—H⋯*A*
N2—H2′⋯O2^i^	0.86 (2)	2.13 (2)	2.8799 (19)	145.9 (18)
N3—H3′⋯N1	0.85 (1)	2.25 (2)	2.6604 (17)	110.0 (14)
O2—H2⋯O3^i^	0.87 (2)	2.28 (2)	2.8867 (16)	127.1 (18)
O2—H2⋯O1	0.87 (2)	2.14 (2)	2.6206 (16)	114.2 (17)

Symmetry code: (i) 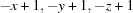.

**Fig. 1.**
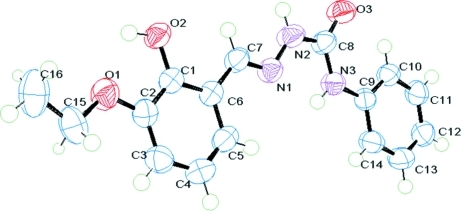
ORTEP diagram of 2-(3-ethoxy-2-hydroxybenzyl)-N-phenylhydrazinecarboxamide with 50% probability ellipsoid.

**Fig. 2.**
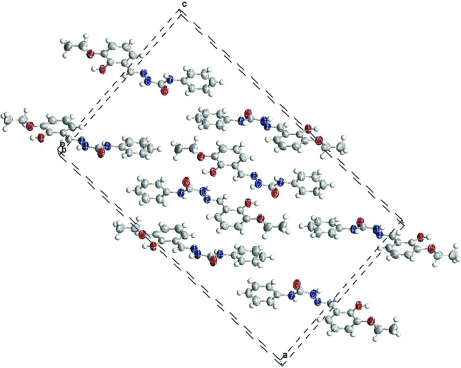
Packing diagram of 2-(3-ethoxy-2-hydroxybenzyl)-N-phenylhydrazinecarboxamide along b axis.

## supplementary crystallographic information

### Comment

The compound crystallizes into a monoclinic space group *C*2/*c*. The molecule is almost planar with maximum deviation of 0.218 (1) Å for the atom N1. The dihedral angle between the two aromatic rings is 6.70°. The molecule exists in the *E* configuration with respect to C7=N1 bond (Fig. 1). A torsion angle value of -176.4 (1)° corresponding to O3–C8–N2–N1 moiety confirms the *trans* configuration of the O3 atom with respect to hydrazine nitrogen atom N1. As a result, the atom N1 lies *cis* to N3, with an N1–N2–C8–N3 torsion angle of 3.6 (2). This arrangement favours the intramolecular hydrogen bond interaction between N1 and H attached to N3 atom. Similarly O1 and O2 lie *cis* to each other with an torsion angle of -0.4 (2) and it favours the intramolecular hydrogen bond interaction between O1 and the H on O2 atom. These two intramolecular hydrogen bonding interactions play an important role by stabilizing this conformation. The C8–N2 bond distance [1.3656 (19) Å] is appreciably close to that of C–N single bond [1.351 (2) Å], confirming the keto form of the ligand (Reena & Kurup, 2010). The existence of 2-(3-ethoxy-2-hydroxybenzyl)-*N*-phenylhydrazinecarboxamide in the keto form in the solid state is evidenced by the C8–O2 bond distance of 1.2233 (19) Å, which is very close to a formal C=O bond length [1.21 Å] (Kala *et al.*, 2007).

The neighbouring molecules are interconnected by intermolecular hydrogen bonding (Table 1). The molecular array involes two types of hydrogen bonding interactions where the O1 and O3 function as acceptors while the atom O2 acts as donor and acceptor.

In the crystal lattice (Fig. 2), two-dimensional packing is fashioned by the network of intermolecular hydrogen bonding interactions. The repeating units of two adjacent molecules are aligned in offset manner. The distance between two consecutive parallel rings is more than 5 Å and therefore there are very weak π···π or C–H···π interactions between the adjacent molecules. However, the hydrogen bonding plays key role in packing of molecules in the unit cell.

### Experimental

The title compound was prepared by adapting a reported procedure (Siji *et al.*, 2010). A methanolic solution (30 ml) of *N*-phenylhydrazinecarboxamide (1.511 g, 10 mmol) was added to a solution of 3-ethoxy-2-hydroxybenzaldehyde (1.662 g, 10 mmol) in methanol and the reaction mixture was refluxed for 2 h after adding a few drops of dilute acetic acid. On cooling the solution, very pale yellow block-shaped crystals suitable for single-crystal analysis were obtained.

### Refinement

All H atoms on C were placed in calculated positions, guided by difference maps, with C—H bond distances 0.93–0.97 Å. H atoms were assigned as *U*_iso_=1.2U_eq_ (1.5 for Me). N3—H3' and O2—H2 H atoms were located from difference maps and restrained using *DFIX* instructions. N2—H2' hydrogen is located from difference maps and was freely refined.

### Crystal data

**Table:**

C_16_H_17_N_3_O_3_	*F*(000) = 1264.0
*M**_r_* = 299.33	*D*_x_ = 1.305 Mg m^−^^3^
Monoclinic, *C*2/*c*	Mo *K*α radiation, λ = 0.71073 Å
Hall symbol: -C 2yc	Cell parameters from 3454 reflections
*a* = 30.1352 (13) Å	θ = 1.4–27.5°
*b* = 5.5552 (3) Å	µ = 0.09 mm^−^^1^
*c* = 18.2232 (8) Å	*T* = 296 K
β = 92.753 (2)°	Block, pale yellow
*V* = 3047.2 (2) Å^3^	0.50 × 0.30 × 0.10 mm
*Z* = 8	

### Data collection

**Table:**

Bruker APEXII CCD diffractometer	2687 independent reflections
Radiation source: fine-focus sealed tube	2066 reflections with *I* > 2σ(*I*)
graphite	*R*_int_ = 0.035
ω and φ scans	θ_max_ = 25.0°, θ_min_ = 2.6°
Absorption correction: multi-scan (*SADABS*; Sheldrick, 1996)	*h* = −35→35
*T*_min_ = 0.967, *T*_max_ = 0.991	*k* = −4→6
10811 measured reflections	*l* = −21→21

### Refinement

**Table:**

Refinement on *F*^2^	Secondary atom site location: difference Fourier map
Least-squares matrix: full	Hydrogen site location: inferred from neighbouring sites
*R*[*F*^2^ > 2σ(*F*^2^)] = 0.040	H atoms treated by a mixture of independent and constrained refinement
*wR*(*F*^2^) = 0.137	*w* = 1/[σ^2^(*F*_o_^2^) + (0.0783*P*)^2^ + 0.6395*P*] where *P* = (*F*_o_^2^ + 2*F*_c_^2^)/3
*S* = 1.06	(Δ/σ)_max_ = 0.003
2687 reflections	Δρ_max_ = 0.16 e Å^−^^3^
213 parameters	Δρ_min_ = −0.13 e Å^−^^3^
2 restraints	Extinction correction: *SHELXL97* (Sheldrick, 1996), Fc^*^=kFc[1+0.001xFc^2^λ^3^/sin(2θ)]^-1/4^
Primary atom site location: structure-invariant direct methods	Extinction coefficient: 0.0055 (9)

### Special details

**Table:**

Geometry. All e.s.d.'s (except the e.s.d. in the dihedral angle between two l.s. planes) are estimated using the full covariance matrix. The cell e.s.d.'s are taken into account individually in the estimation of e.s.d.'s in distances, angles and torsion angles; correlations between e.s.d.'s in cell parameters are only used when they are defined by crystal symmetry. An approximate (isotropic) treatment of cell e.s.d.'s is used for estimating e.s.d.'s involving l.s. planes.
Refinement. Refinement of *F*^2^ against ALL reflections. The weighted *R*-factor *wR* and goodness of fit *S* are based on *F*^2^, conventional *R*-factors *R* are based on *F*, with *F* set to zero for negative *F*^2^. The threshold expression of *F*^2^ > σ(*F*^2^) is used only for calculating *R*-factors(gt) *etc*. and is not relevant to the choice of reflections for refinement. *R*-factors based on *F*^2^ are statistically about twice as large as those based on *F*, and *R*- factors based on ALL data will be even larger.

### Fractional atomic coordinates and isotropic or equivalent isotropic displacement parameters (Å^2^)

**Table:**

	*x*	*y*	*z*	*U*_iso_*/*U*_eq_	
O1	0.37161 (4)	−0.1876 (2)	0.50043 (7)	0.0781 (4)	
O2	0.42932 (4)	0.1621 (2)	0.48991 (6)	0.0703 (4)	
O3	0.61791 (4)	0.7085 (2)	0.64595 (7)	0.0800 (4)	
N1	0.53666 (4)	0.2594 (3)	0.62904 (7)	0.0621 (4)	
N2	0.56032 (5)	0.4651 (3)	0.61776 (8)	0.0726 (4)	
N3	0.61370 (4)	0.3406 (2)	0.70231 (8)	0.0626 (4)	
C1	0.43561 (5)	0.0007 (3)	0.54552 (8)	0.0563 (4)	
C2	0.40572 (5)	−0.1888 (3)	0.55286 (9)	0.0611 (4)	
C3	0.41199 (6)	−0.3509 (3)	0.60918 (10)	0.0719 (5)	
H3	0.3924	−0.4784	0.6139	0.086*	
C4	0.44794 (6)	−0.3228 (3)	0.65907 (10)	0.0773 (5)	
H4	0.4521	−0.4315	0.6976	0.093*	
C5	0.47737 (6)	−0.1374 (3)	0.65230 (9)	0.0691 (5)	
H5	0.5013	−0.1219	0.6861	0.083*	
C6	0.47181 (5)	0.0285 (3)	0.59510 (8)	0.0567 (4)	
C7	0.50174 (5)	0.2319 (3)	0.58751 (8)	0.0616 (4)	
H7	0.4952	0.3456	0.5511	0.074*	
C8	0.59928 (5)	0.5163 (3)	0.65588 (9)	0.0616 (4)	
C9	0.65140 (5)	0.3413 (3)	0.75132 (8)	0.0542 (4)	
C10	0.68369 (5)	0.5188 (3)	0.75262 (9)	0.0626 (4)	
H10	0.6812	0.6481	0.7203	0.075*	
C11	0.71966 (5)	0.5025 (3)	0.80230 (10)	0.0691 (5)	
H11	0.7413	0.6217	0.8030	0.083*	
C12	0.72396 (6)	0.3148 (3)	0.85037 (10)	0.0743 (5)	
H12	0.7484	0.3057	0.8834	0.089*	
C13	0.69195 (7)	0.1402 (3)	0.84939 (11)	0.0818 (6)	
H13	0.6946	0.0122	0.8822	0.098*	
C14	0.65571 (6)	0.1520 (3)	0.80001 (10)	0.0702 (5)	
H14	0.6342	0.0319	0.7997	0.084*	
C15	0.33793 (6)	−0.3673 (4)	0.50345 (11)	0.0823 (6)	
H15A	0.3258	−0.3702	0.5518	0.099*	
H15B	0.3502	−0.5248	0.4935	0.099*	
C16	0.30266 (7)	−0.3044 (5)	0.44677 (14)	0.1099 (8)	
H16A	0.2900	−0.1514	0.4584	0.165*	
H16B	0.2799	−0.4256	0.4458	0.165*	
H16C	0.3153	−0.2956	0.3995	0.165*	
H2	0.4079 (6)	0.112 (4)	0.4600 (11)	0.093 (6)*	
H3'	0.5986 (5)	0.2112 (18)	0.7011 (10)	0.074 (5)*	
H2'	0.5514 (7)	0.570 (4)	0.5859 (12)	0.096 (7)*	

### Atomic displacement parameters (Å^2^)

**Table:**

	*U*^11^	*U*^22^	*U*^33^	*U*^12^	*U*^13^	*U*^23^
O1	0.0642 (7)	0.0915 (9)	0.0775 (8)	−0.0189 (6)	−0.0083 (6)	0.0033 (6)
O2	0.0683 (7)	0.0755 (8)	0.0652 (7)	−0.0102 (6)	−0.0178 (6)	0.0151 (6)
O3	0.0740 (7)	0.0724 (8)	0.0912 (9)	−0.0133 (6)	−0.0211 (6)	0.0243 (7)
N1	0.0565 (7)	0.0674 (8)	0.0616 (8)	−0.0023 (6)	−0.0046 (6)	0.0058 (6)
N2	0.0641 (8)	0.0762 (10)	0.0754 (9)	−0.0100 (7)	−0.0171 (7)	0.0225 (8)
N3	0.0582 (7)	0.0607 (8)	0.0676 (8)	−0.0071 (6)	−0.0094 (6)	0.0085 (7)
C1	0.0559 (8)	0.0610 (9)	0.0521 (8)	0.0027 (7)	0.0015 (6)	0.0008 (7)
C2	0.0578 (8)	0.0666 (10)	0.0591 (9)	−0.0032 (8)	0.0058 (7)	−0.0045 (8)
C3	0.0784 (11)	0.0679 (11)	0.0700 (10)	−0.0114 (9)	0.0102 (9)	0.0017 (8)
C4	0.0940 (13)	0.0763 (12)	0.0616 (10)	−0.0024 (10)	0.0035 (9)	0.0163 (9)
C5	0.0743 (10)	0.0764 (11)	0.0558 (9)	0.0008 (9)	−0.0055 (8)	0.0073 (8)
C6	0.0573 (8)	0.0618 (9)	0.0508 (8)	0.0019 (7)	0.0007 (6)	0.0000 (7)
C7	0.0592 (8)	0.0702 (10)	0.0546 (8)	−0.0008 (8)	−0.0053 (7)	0.0076 (7)
C8	0.0572 (8)	0.0668 (10)	0.0600 (9)	−0.0011 (8)	−0.0045 (7)	0.0079 (8)
C9	0.0538 (8)	0.0535 (8)	0.0551 (8)	0.0036 (7)	−0.0007 (6)	−0.0019 (7)
C10	0.0634 (9)	0.0609 (9)	0.0627 (9)	−0.0027 (7)	−0.0062 (7)	0.0058 (7)
C11	0.0637 (9)	0.0672 (11)	0.0751 (10)	−0.0052 (8)	−0.0108 (8)	−0.0029 (8)
C12	0.0738 (10)	0.0710 (11)	0.0758 (11)	0.0095 (9)	−0.0216 (9)	−0.0042 (9)
C13	0.0960 (13)	0.0626 (11)	0.0844 (12)	0.0049 (10)	−0.0221 (10)	0.0147 (9)
C14	0.0728 (10)	0.0567 (10)	0.0796 (11)	−0.0039 (8)	−0.0104 (9)	0.0088 (8)
C15	0.0652 (10)	0.0861 (13)	0.0963 (14)	−0.0172 (9)	0.0108 (9)	−0.0240 (11)
C16	0.0634 (11)	0.157 (2)	0.1087 (16)	−0.0202 (13)	−0.0018 (11)	−0.0364 (16)

### Geometric parameters (Å, °)

**Table:**

O1—C2	1.3693 (19)	C5—H5	0.9300
O1—C15	1.426 (2)	C6—C7	1.456 (2)
O2—C1	1.3598 (18)	C7—H7	0.9300
O2—H2	0.870 (15)	C9—C14	1.378 (2)
O3—C8	1.2239 (19)	C9—C10	1.385 (2)
N1—C7	1.2758 (19)	C10—C11	1.381 (2)
N1—N2	1.368 (2)	C10—H10	0.9300
N2—C8	1.365 (2)	C11—C12	1.364 (3)
N2—H2'	0.86 (2)	C11—H11	0.9300
N3—C8	1.350 (2)	C12—C13	1.368 (3)
N3—C9	1.4109 (19)	C12—H12	0.9300
N3—H3'	0.8500 (11)	C13—C14	1.383 (2)
C1—C6	1.391 (2)	C13—H13	0.9300
C1—C2	1.396 (2)	C14—H14	0.9300
C2—C3	1.371 (2)	C15—C16	1.488 (3)
C3—C4	1.389 (2)	C15—H15A	0.9700
C3—H3	0.9300	C15—H15B	0.9700
C4—C5	1.369 (2)	C16—H16A	0.9600
C4—H4	0.9300	C16—H16B	0.9600
C5—C6	1.396 (2)	C16—H16C	0.9600
			
C2—O1—C15	118.77 (14)	N3—C8—N2	114.31 (15)
C1—O2—H2	109.1 (15)	C14—C9—C10	119.20 (15)
C7—N1—N2	115.60 (13)	C14—C9—N3	116.98 (14)
C8—N2—N1	122.63 (14)	C10—C9—N3	123.82 (14)
C8—N2—H2'	116.0 (14)	C11—C10—C9	119.55 (15)
N1—N2—H2'	121.4 (14)	C11—C10—H10	120.2
C8—N3—C9	128.31 (14)	C9—C10—H10	120.2
C8—N3—H3'	116.1 (12)	C12—C11—C10	121.22 (16)
C9—N3—H3'	115.5 (12)	C12—C11—H11	119.4
O2—C1—C6	119.17 (14)	C10—C11—H11	119.4
O2—C1—C2	120.09 (13)	C11—C12—C13	119.26 (16)
C6—C1—C2	120.74 (14)	C11—C12—H12	120.4
O1—C2—C3	126.67 (15)	C13—C12—H12	120.4
O1—C2—C1	113.30 (14)	C12—C13—C14	120.60 (16)
C3—C2—C1	120.02 (15)	C12—C13—H13	119.7
C2—C3—C4	119.42 (16)	C14—C13—H13	119.7
C2—C3—H3	120.3	C9—C14—C13	120.16 (16)
C4—C3—H3	120.3	C9—C14—H14	119.9
C5—C4—C3	120.92 (16)	C13—C14—H14	119.9
C5—C4—H4	119.5	O1—C15—C16	107.15 (18)
C3—C4—H4	119.5	O1—C15—H15A	110.3
C4—C5—C6	120.61 (16)	C16—C15—H15A	110.3
C4—C5—H5	119.7	O1—C15—H15B	110.3
C6—C5—H5	119.7	C16—C15—H15B	110.3
C1—C6—C5	118.29 (15)	H15A—C15—H15B	108.5
C1—C6—C7	119.64 (13)	C15—C16—H16A	109.5
C5—C6—C7	122.04 (14)	C15—C16—H16B	109.5
N1—C7—C6	122.26 (14)	H16A—C16—H16B	109.5
N1—C7—H7	118.9	C15—C16—H16C	109.5
C6—C7—H7	118.9	H16A—C16—H16C	109.5
O3—C8—N3	125.98 (14)	H16B—C16—H16C	109.5
O3—C8—N2	119.71 (15)		
			
C7—N1—N2—C8	−177.55 (15)	C1—C6—C7—N1	−176.32 (14)
C15—O1—C2—C3	1.3 (3)	C5—C6—C7—N1	5.7 (2)
C15—O1—C2—C1	−177.82 (14)	C9—N3—C8—O3	3.1 (3)
O2—C1—C2—O1	−0.5 (2)	C9—N3—C8—N2	−177.15 (15)
C6—C1—C2—O1	178.87 (13)	N1—N2—C8—O3	−176.50 (15)
O2—C1—C2—C3	−179.69 (15)	N1—N2—C8—N3	3.7 (2)
C6—C1—C2—C3	−0.4 (2)	C8—N3—C9—C14	171.60 (17)
O1—C2—C3—C4	−178.35 (16)	C8—N3—C9—C10	−8.7 (3)
C1—C2—C3—C4	0.8 (3)	C14—C9—C10—C11	0.3 (2)
C2—C3—C4—C5	−0.7 (3)	N3—C9—C10—C11	−179.42 (14)
C3—C4—C5—C6	0.2 (3)	C9—C10—C11—C12	−0.2 (3)
O2—C1—C6—C5	179.23 (14)	C10—C11—C12—C13	−0.2 (3)
C2—C1—C6—C5	−0.1 (2)	C11—C12—C13—C14	0.4 (3)
O2—C1—C6—C7	1.2 (2)	C10—C9—C14—C13	−0.1 (3)
C2—C1—C6—C7	−178.15 (13)	N3—C9—C14—C13	179.66 (16)
C4—C5—C6—C1	0.2 (2)	C12—C13—C14—C9	−0.3 (3)
C4—C5—C6—C7	178.16 (16)	C2—O1—C15—C16	172.82 (16)
N2—N1—C7—C6	−176.88 (14)		

### Hydrogen-bond geometry (Å, °)

**Table:**

*D*—H···*A*	*D*—H	H···*A*	*D*···*A*	*D*—H···*A*
N2—H2'···O2^i^	0.86 (2)	2.13 (2)	2.8799 (19)	145.9 (18)
N3—H3'···N1	0.85 (1)	2.25 (2)	2.6604 (17)	110.(1)
O2—H2···O3^i^	0.87 (2)	2.28 (2)	2.8867 (16)	127.(2)
O2—H2···O1	0.87 (2)	2.14 (2)	2.6206 (16)	114.(2)

Symmetry codes: (i) −*x*+1, −*y*+1, −*z*+1.
